# Psychosocial and economic impact of COVID-19 pandemic by sex among migrant populations compared with general Finnish population: a population-based study

**DOI:** 10.1177/14034948241235245

**Published:** 2024-03-27

**Authors:** Sanna Nykänen, Regina García-Velázquez, Anu E Castaneda, Päivikki Koponen, Laura Musta, Natalia Skogberg

**Affiliations:** 1 Department of Public Health and Welfare, Equality Unit, Finnish Institute for Health and Welfare, Helsinki, Finland; 2 Department of Public Health and Welfare, Finnish Institute for Health and Welfare, Helsinki, Finland

**Keywords:** Migration, COVID-19, psychosocial, economic, wellbeing

## Abstract

**Aims:**

To study sex differences in the psychosocial and economic impact of the restrictive measures during the COVID-19 pandemic in 2020 among the migrant origin and the general population in Finland.

**Methods:**

Cross-sectional MigCOVID Survey data (10/2020–2/2021; *n*=3668) were used. FinHealth 2017 Follow-up Study participants constituted the general population reference group (*n*=3490). Sex differences in self-perceived impact of the restrictive measures during the COVID-19 pandemic in 2020 on the psychosocial and economic situation were examined with multivariate logistic regression, adjusting for sociodemographics and self-rated health.

**Results:**

The migrant origin population had higher odds for reporting weakened economic situation (odds ratio (OR) 5.41; 95% confidence interval (CI) 3.96–7.39), increased loneliness (OR 1.75; 95% CI 1.35–2.28), decrease in feelings of hope for the future (OR 1.70; 95% CI 1.33–2.19) and increased sleeping difficulties and nightmares (OR 1.98; 95% CI 1.34–2.92) than the general population. While the psychosocial and economic impact of COVID-19 was higher in women compared with men in the general population, findings were not fully replicated in the migrant origin population.

**Conclusions:**

**Individuals of migrant origin faced a higher likelihood of experiencing adverse changes in both psychosocial and economic aspects during the pandemic, suggesting increased vulnerability linked to migrant origin. Additional research is required to delve into the intricate connections among gender, migrant origin, and the impact of COVID-19, aiming to enhance comprehensive understanding of the contributing factors. Vulnerabilities of different population groups should be identified and addressed when planning measures to reduce adverse societal impact in future crises.**

## Background

A growing body of evidence shows that restrictive measures during COVID-19 had a disproportionally higher impact on the migrant origin populations’ health and wellbeing compared with general populations in the European region [1, 2]. In Finland the first COVID-19 case was reported in January 2020 and remained single until the end of February when it began spreading in the population. In response, the government submitted a decree to parliament in March 2020, implementing the Emergency Powers Act. Stringent measures, such as closing educational and recreational facilities, prohibiting gatherings of over 10 people, and urging public avoidance, were introduced [3]. The restrictive measures especially affected those individuals in vulnerable positions. Migrant origin populations have, for example, experienced higher rates of hospitalisation and morbidity and mortality compared with the general population in Finland during the COVID-19 pandemic [3, 4].

Although population-based data on the psychosocial impact of restrictive measures during COVID-19 on migrant origin populations are limited, several previous studies have reported higher rates of psychological distress such as depression, anxiety and sleep disturbances, adverse impact on health and wellbeing and greater barriers in access to risk communications compared with general populations [3, 5]. Deterioration in the economic situation and health have also been reported [6].

Concerns have also been raised on the particularly high impact of restrictive measures during COVID-19 among migrant origin women. The United Nations (UN) policy brief on the impact of COVID-19 on women stressed the importance of considering different intersecting factors, such as age, sex and background in political planning and decision making [7]. Only a handful of studies on the impact of COVID-19 and its restrictive measures among migrant origin populations have used sex as one of the background variables, or have compared the results among migrant origin women with women in the general population [8]. The impact of restrictive measures during COVID-19 has been explored more extensively in general population samples, showing greater deterioration in the economic situation and higher rates of psychological distress in women compared with men [9–11]. COVID-19 and its restrictive measures have been suggested to exacerbate these gendered inequalities further.

While gendered inequalities have been raised as an important point of concern during COVID-19, there is a gaping lack in studies focusing specifically on exploring the extent of these inequalities and associated factors in migrant origin populations, especially in the European context. Information on the impact of restrictive measures during COVID-19 by sex among migrant origin populations is acutely needed to deepen our understanding and to plan restorative measures better, as well as to improve preparedness for future crises.

This study addresses the gap in knowledge on sex differences in the self-perceived impact on psychosocial (contact with friends and relatives, loneliness, disputes and conflicts within the family, hope for the future, and sleeping difficulties, nightmares) and the economic impact of restrictive measures of COVID-19 among migrant origin populations, by comparing a nationally representative sample of migrant origin men and women with men and women in the general Finnish population. Advancing this knowledge is important for addressing and implementing effective evidence-based migrant-sensitive policies and strategies to ensure equality in health and wellbeing for all.

## Methods

### Study participants

This cross-sectional study is based on the data from the Impact of the Coronavirus on the Wellbeing of the Foreign born Population (MigCOVID) Survey (10/2020–2/2021; *n*=3668), with participants in the FinHealth 2017 Follow-up Study as the general population reference group (*n*=3490) [12, 13]. The MigCOVID Survey is a follow-up study to the Survey on Wellbeing among Foreign Born Population (FinMonik) [14], conducted between 2018 and 2019 [15]. For the original FinMonik Survey, a stratified random sample of 13,650 persons was drawn from the Finnish Population Register in 2018. The inclusion criteria were that the person was themselves born abroad, both parents or the only known parent were born abroad or the country of birth was unknown, age 18–64 years, and length of residence in Finland at least one year. Altogether 6836 persons participated in the FinMonik Survey. Participants in the FinMonik Survey, who gave permission for further contact and still lived in Finland in 2020 when the original sample was updated, were invited to participate in the MigCOVID Survey (*n*=5259). Participants in the MigCOVID Survey were aged 20–66 years. A supplementary sample (*n*=982) of migrant origin persons born in Somalia was drawn from the Finnish Population Register in 2020. The participation rate for the MigCOVID Survey was 60% out of those who were invited to participate in this follow-up study.

The FinHealth 2017 Follow-up Study was conducted within the same time frame as the MigCOVID Survey and followed a comparative study protocol. The original sample of the FinHealth 2017 Study constituted a random sample drawn in 2017 from the Finnish Population Register of persons aged 18 years and older (*n*=10,305). Those who declined any further contact during the original FinHealth 2017 Study, and those who were no longer living in Finland were excluded from the follow-up survey. Otherwise, the whole original sample was invited to take part in the FinHealth 2017 Follow-up Study in 2020 (*n*=9580) with a participation rate of 53%. A sub-sample of FinHealth 2017 Follow-up Study participants aged 21–66 years was used in the current study to match the age range of the MigCOVID Survey participants.

### Variables

The psychosocial factors in this study were: (a) contact with friends and relatives; (b) loneliness; (c) disputes and conflicts within the family; (d) hope for the future; and (e) sleeping difficulties, nightmares. These five psychosocial factors were collected from a wider battery of items with the same statement: ‘Has the corona epidemic or its restrictive measures affected your everyday life?’ with a same response scale of: ‘no effect’, ‘yes, increased’, ‘yes, decreased’ and ‘does not apply’. For this study, the responses ‘no effect’ and ‘does not apply’ were combined. Economic situation was based on the item ‘Has the corona epidemic weakened your financial situation?’ and rated with the following five-point scale: ‘very much’, ‘quite a lot’, ‘to some extent’, ‘a little’ and ‘not at all’. For this study, the variable was dichotomised combining ‘very much’ and ‘quite a lot’ to classify experiences of weakened economic situation. Psychosocial and economic factors included in this study were selected based on a literature review and the availability of measures included in the survey questionnaire.

The sociodemographic and health-related characteristics and their categorisations can be found in Table I. Information on age, sex, and length of residence in Finland were obtained from the Finnish Population Register. Country group was collected from registers for the MigCOVID participants only, and was coded into four categories based on the country of birth of the participants: Russia and the former Soviet Union; rest of Europe, America and Oceania; Africa and Middle East; and Asia and Latin America, meaning all southeast Asian, east Asian, south and central Asian and Latin American countries. Measures on the highest completed level of education in Finland or abroad, economic activity and Finnish and Swedish (official languages of Finland) language proficiency were based on self-report.

**Table I.** Sociodemographic characteristics of the sample, men and women, weighted *n* %.

Men	Russia, former Soviet Union, *n*=290 (%)	Rest of Europe, North America and Oceania, *n*=632 (%)	Africa and the Middle East, *n*=483 (%)	Asia and Latin America, *n*=281 (%)	Born abroad total, men *N*=1641 (%)	General population total, men, *N*=1814 (%)
Age, years						
20–34	91 (31.4)	148 (23.4)	162 (37)	119 (42.3)	520 (31.7)	493 (27.2)
35–49	127 (43.7)	285 (45.1)	181 (41.4)	102 (36.2)	696 (42.2)	627 (34.5)
50–66	72 (24.8)	199 (31.5)	95 (21.7)	60 (21.2)	426 (25.9)	694 (38.3)
Education						
Primary education or less	21 (7.2)	69 (11)	51 (11.8)	31 (11.1)	171 (10.6)	137 (7.7)
Secondary education	128 (44.7)	257 (41)	188 (43.8)	83 (29.1)	657 (40.4)	969 (54.5)
Tertiary or higher education	138 (48.1)	302 (48.1)	191 (44.3)	166 (59.3)	796 (49)	670 (37.8)
Living alone						
Yes	89 (30.8)	173 (27.4)	122 (27.9)	42 (14.9)	427 (26)	522 (28.8)
No	200 (69.2)	459 (72.6)	316 (72.1)	239 (85.1)	1215 (74)	1292 (71.2)
Economic activity						
Working full time/part time	211 (74.1)	464 (75)	262 (60.7)	191 (68.4)	1128 (69.9)	1350 (75.1)
Student	12 (4.2)	16 (2.5)	57 (13.2)	21 (7.5)	106 (6.5)	129 (7.2)
Other	62 (21.7)	139 (22.4)	112 (26)	67 (24.1)	380 (23.5)	319 (17.8)
Language skills (Finnish or Swedish)						
Intermediate or less	172 (59.8)	360 (57.7)	257 (59.8)	215 (77.3)	1003 (62)	0 (0)
Proficient	166 (40.2)	263 (42.3)	172 (40.2)	63 (22.7)	615 (38)	0 (0)
Length of residence in Finland, years						
3–6	35 (17.1)	82 (12.9)	104 (23.7)	66 (23.5)	286 (17.4)	0 (0)
7–11	56 (19.3)	223 (35.3)	131 (29.9)	89 (31.7)	499 (30.4)	0 (0)
12+	199 (68.8)	327 (51.7)	204 (46.4)	126 (44.8)	856 (52.1)	0 (0)
Self-reported health						
Good or quite good	187 (64.7)	485 (77.1)	231 (52.8)	109 (39)	1201 (73.4)	1445 (80.1)
Average or worse	102 (35.3)	145 (22.9)	206 (47.2)	171 (61)	435 (26.6)	360 (19.9)
Women	Russia, former Soviet Union, *n*=464, (%)	Rest of Europe, North America and Oceania, *n*=457, (%)	Africa and the Middle East, *n*=237, (%)	Asia and Latin America, *n*=369, (%)	Born abroad total, women, *N*=1562, (%)	General population, women, *N*=1780, (%)
Age, years						
20–34	105 (22.6)	142 (31.2)	111 (40.8)	116 (31.4)	474 (30.3)	493 (27.7)
35–49	170 (36.7)	181 (39.7)	102 (37.6)	169 (45.8)	623 (39.9)	602 (33.8)
50–66	189 (40.7)	133 (29.1)	59 (21.6)	84 (22.8)	465 (29.8)	685 (38.5)
Education						
Primary education or less	14 (3.1)	60 (13.5)	37 (13.9)	37 (10)	147 (9.7)	84 (4.8)
Secondary education	184 (41.3)	155 (34.7)	127 (47.7)	124 (34.2)	590 (38.8)	788 (44.8)
Tertiary or higher education	249 (55.7)	230 (51.8)	102 (38.5)	203 (55.8)	784 (51.6)	885 (50.4)
Living alone						
Yes	88 (19)	68 (15)	28 (10.3)	23 (6.2)	207 (13.3)	412 (23.1)
No	376 (81)	388 (85)	245 (89.7)	346 (93.8)	1354 (86.7)	1368 (76.9)
Economic activity						
Working full time/part time	290 (64.2)	284 (64)	157 (60.3)	237 (67.5)	969 (63.9)	1168 (67.7)
Student	34 (7.6)	38 (8.3)	42 (16)	32 (9.1)	146 (9.6)	169 (9.7)
Other	127 (28.2)	130 (28.7)	62 (23.7)	82 (23.4)	401 (26.5)	396 (22.6)
Language skills (Finnish or Swedish)						
Intermediate or less	199 (43.9)	216 (47.8)	138 (53.4)	266 (72.5)	819 (53.5)	0 (0)
Proficient	255 (56.1)	236 (52.2)	120 (46.6)	101 (27.5)	712 (46.5)	0 (0)
Length of residence in Finland, years						
3–6	39 (8.4)	87 (19.2)	49 (17.9)	105 (28.4)	280 (17.9)	0 (0)
7–11	79 (17)	155 (33.9)	62 (22.6)	110 (29.8)	405 (25.9)	0 (0)
12+	346 (74.6)	214 (46.9)	162 (59.6)	154 (41.9)	877 (56.2)	0 (0)
Self-reported health						
Good or quite good	186 (40.2)	310 (68.8)	188 (69.1)	262 (71.5)	1094 (70.5)	1361 (77.6)
Average or worse	277 (59.8)	141 (31.2)	84 (30.9)	105 (28.2)	458 (29.5)	392 (22.4)

### Statistical analyses

Prevalence rates and their 95% confidence intervals were estimated adjusting for age. The bivariate association between the six psychosocial factors with sample and sex (within sample) was inspected by means of cross-tables and tested with chi-square tests. The *P* values obtained were adjusted for multiple comparison [16] and effect sizes computed according to Cramer’s *v* [17] following the formula χ2(n×df) , where χ^2^ is the chi-square statistic and df is the minimum number of categories minus one. In this case df=1, because both sample and sex were coded as binary variables. We considered the effect size to be small when surpassing 0.06, to be medium to moderate when at least 0.17, and to be large when reaching 0.30 [18].

Finally, we conducted a series of stepwise logistic regression models, one for every outcome variable at a time. The event modelled, a binary variable (increase, decrease), was ‘changed to worse as consequence of COVID-19 pandemic’ (i.e. increase in disputes and conflicts within the family, or decreased hopes for the future). We added blocks of predictors to those already included in earlier steps of the modelling process. In model 1 (Supplemental Table I), we included the following predictors: sample (foreign-born/general population), sex, sample*sex interaction, and age group. In model 2 (Supplemental Table II) we then added educational level and economic activity. In model 3 (Table II), self-reported health and living alone at household were finally added. All analyses were conducted using R software [19] and the package survey [20]. The stratified random sampling structure of the data was accounted for in all stages of data analysis by using sample weights and their stratum structure.

**Table II.** The association of sociodemographic, employment and health-related characteristics with studied psychosocial and economic impact during COVID-19, adjusted for sociodemographic factors,^a^ logistic regression model estimates for model 3.

Model 3.	Decrease in contact with friends and relatives OR (95% CI) *P* value	Increase in loneliness OR (95% CI) *P* value	Increase in disputes and conflicts within the family OR (95% CI) *P* value	Decrease in hope for the future OR (95% CI) *P* value	Increase in sleeping difficulties, nightmares OR (95% CI) *P* value	Weakening of the financial situation OR (95% CI) *P* value
Sample						
General	Ref.	Ref.	Ref.	Ref.	Ref.	Ref.
Foreign	1.01 (0.79–1.29)	1.75 (1.35–2.28)**	1.19 (0.85–1.66)	1.70 (1.33–2.19)**	1.98 (1.34–2.92)**	5.41 (3.96–7.39)**
Sex						
Male	Ref.	Ref.	Ref.	Ref.	Ref.	Ref.
Female	1.13 (0.91–1.39)	1.86 (1.46–2.37)**	1.08 (0.81–1.46)	1.57 (1.25–1.96)**	1.46 (1.06–2.03)*	1.18 (0.84–1.65)
Sample × sex						
Foreign female	0.82 (0.60–1.13)	0.63 (0.45–0.88)*	0.90 (0.58–1.39)	0.65 (0.47–0.90)*	1.01 (0.63–1.60)	0.61 (0.39–0.95)*
Age						
20–34	Ref.	Ref.	Ref.	Ref.	Ref.	Ref.
35–49	1.08 (0.87–1.35)	0.83 (0.66–1.04)	0.90 (0.68–1.20)	0.90 (0.72–1.12)	0.98 (0.72–1.35)	0.89 (0.67–1.18)
50–66	1.25 (0.99–1.58)	0.58 (0.46–0.73)**	0.58 (0.43–0.78)**	0.96 (0.77–1.20)	0.88 (0.63–1.22)	0.51 (0.38–0.70)**
Education						
Basic level or less	Ref.	Ref.	Ref.	Ref.	Ref.	Ref.
Secondary	1.57 (1.19–2.06)**	0.95 (0.69–1.30)	1.34 (0.86–2.08)	1.37 (1.02–1.83)*	1.34 (0.88–2.05)	1.26 (0.82–1.94)
Higher	2.37 (1.79–3.12)**	1.89 (1.38–2.57)**	1.82 (1.18–2.82)*	2.10 (1.57–2.81)**	1.97 (1.32–2.93)**	1.01 (0.65–1.55)
Living alone						
No	Ref.	Ref.	Ref.	Ref.	Ref.	Ref.
Yes	0.67 (0.55–0.81)**	1.66 (1.36–2.04)**	0.48 (0.34–0.68)**	1.12 (0.91–1.39)	1.60 (1.20–2.14)**	0.93 (0.70–1.24)
Economic activity						
Working	Ref.	Ref.	Ref.	Ref.	Ref.	Ref.
Student	0.68 (0.46–1.02)	1.04 (0.69–1.55)	0.90 (0.58–1.38)	1.05 (0.75–1.49)	1.34 (0.78–2.30)	1.74 (1.14–2.65)
Other	0.95 (0.79–1.14)	1.31 (1.07–1.60)*	0.99 (0.76–1.30)	0.91 (0.75–1.10)	1.48 (1.11–1.98)*	3.08 (2.34–4.06)**
Self-reported health						
Good or quite good	Ref.	Ref.	Ref.	Ref.	Ref.	Ref.
Average or worse	1.15 (0.96–1.38)	2.05 (1.72–2.45)**	1.67 (1.31–2.12)**	1.80 (1.51–2.15)**	3.13 (2.46–3.97)**	1.53 (1.17–1.99)*

OR: odds ratio; CI: confidence interval; Ref.: reference group.

Results are reported as weighted values.

* *P*<0.05; **P<0.001.

a Model 3: adjusted with sex, age, education, living situation, economic activity and self-reported health.

### Ethical approval

Ethical permission for the MigCOVID Survey was received from the ethical committee of Terveyden ja hyvinvoinnin laitos (THL) (THL/4061/6.02.01/2020). The ethics committee at the Helsinki and Uusimaa Hospital Region (HUS/2391/2020) approved the FinHealth 2017 Follow-up Study. Participation in the survey was voluntary. By participating, participants permitted the use of their personal information according to the data protection notification on handling personal data.

## Results

Participants’ sociodemographic characteristics are shown in Table I. Economic activity in the migrant origin population (working full time or part time) was almost at the same level as in the general population men and women. The proportion of those with tertiary or higher education was higher in the migrant origin than men in the general population. Women in the migrant origin population were as highly educated as women in the general population. Language skills were more proficient in migrant origin women than men.

**Figure 1.**
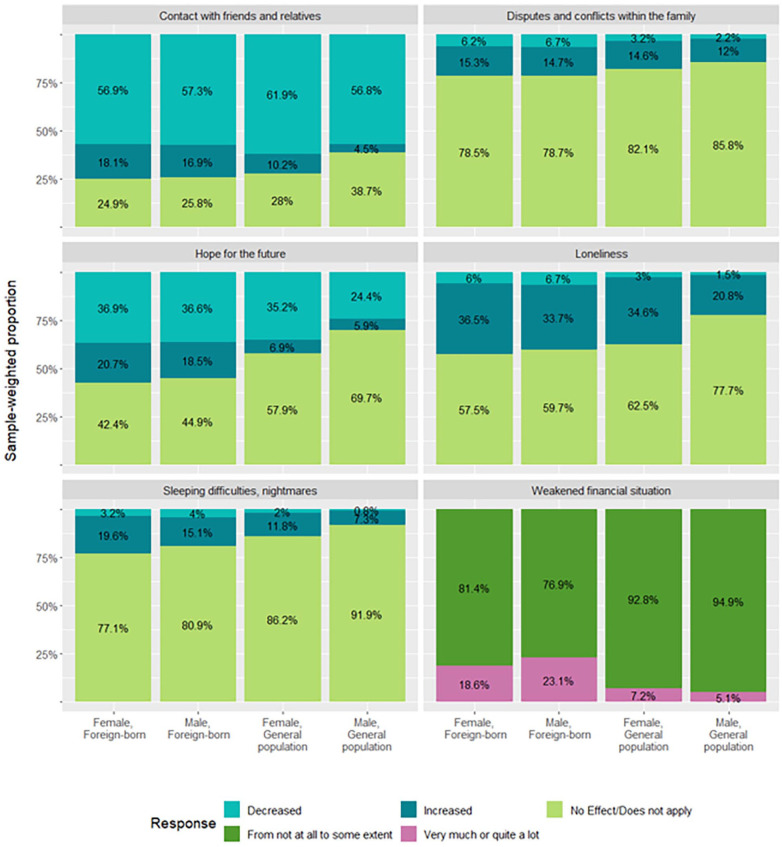
Distribution (%) of the impact of COVID-19 on psychosocial and economic factors among migrant origin and general population men and women.

The sample-weighted, age-adjusted distribution of self-reported changes in psychosocial and economic factors are presented in Figure 1. The overall distribution of psychosocial and economic factors differed among the migrant origin and the general population in Finland (all corrected *P* values <0.01). The general trend was that both men and women of migrant origin reported adverse changes in the psychosocial and economic impact of COVID-19 more frequently than men and women in the general population. When examining sex differences within the migrant origin population and the general population separately, only three factors showed statistically significant differences. In the general population, women reported more disadvantageous changes than men in terms of a decrease in the frequency of contact with friends and relatives (Cramer’s *v* 0.14), increased feelings of loneliness (*v* 0.17) and a decrease in feelings of hope for the future (*v* 0.13). There were no differences between migrant origin men and women in the distribution of any of the studied psychosocial and economic factors.

In Supplemental Table I, a more detailed distribution of these by region of origin is presented. Especially individuals originating from African, Middle Eastern and Asian and Latin American countries reported more adverse psychosocial and economic impacts of COVID-19 compared with the general population. Almost eight out of 10 women of African and Middle Eastern origin reported that their economic situation has weakened during COVID-19, while one out of 10 women in the general population reported the same.

The regression models directly addressed the role of sex and background while adjusting for multiple socioeconomic characteristics. Results of the regression model on step 3 are presented in Table II. Additional tables for regression models step 1 and step 2 are presented in the Supplemental data, Supplemental Table I and Supplemental Table II.

Migrant origin individuals were significantly more likely to report an increase in sleeping difficulties and nightmares (odds ratio (OR) 1.98) as result of the COVID-19 pandemic than the general population. Female sex was also directly associated with reporting more sleeping difficulties and nightmares than men (OR 1.46). Population background and sex together showed a moderating effect over three psychosocial and economic factors: increased feelings of loneliness; decrease in feelings of hope for the future; and worsening of the economic situation (ORs ranging from 0.61 to 0.65). For the migrant origin population, sex differences were smaller or not observed compared with the general population. This result implies that the differences in sex, the trend being more adverse for women, were not constant when comparing the migrant origin population with the general population. In average terms (i.e. main effects), migrant origin background increased the odds of reporting disadvantaged changes in all three psychosocial and economic outcomes by a factor of OR 1.75, OR 1.70 and OR 5.41, respectively. Being a woman additionally increased the odds of feeling lonelier (OR 1.86), having a decrease in hopes for the future (OR 1.57), and sleeping difficulties and nightmares (OR 1.46).

Age showed some associations with psychosocial and economic factors. When compared with the youngest age group (20–24 years), adults aged 50–66 years were less likely to report weakening of their economic situation (OR 0.51), felt less lonely (OR 0.58), and reported fewer disputes and conflicts within the family (OR 0.58).

Educational attainment showed some dose–response associations with few psychosocial and economic factors. The higher the educational attainment, the more likely the participant was to report a decrease in social contacts with friends and relatives (ORs 1.57 and 2.37) and a decrease in feelings of hope for the future (ORs 1.37 and 2.10). Compared with those with a basic educational level or less, highly educated individuals reported being lonelier (OR 1.89), having more disputes and conflicts within the family (OR 1.82), and more sleeping difficulties and nightmares (OR 1.97).

Individuals living alone reported fewer difficulties with keeping social contact with friends and relatives (OR 0.67), increased loneliness (OR 1.66), less conflict (OR 0.48), and more sleeping difficulties and nightmares (OR 1.60) than those who had cohabitants.

Work was, in general, a protective factor against the negative impact of the COVID-19 pandemic. Worsening of the economic situation was reported more often by students (OR 1.74) compared with those who worked any amount of time, full time or part time. Individuals other than workers and students were more likely to report worsening of the economic situation (OR 3.08), increased loneliness (OR 1.31), and sleeping difficulties and nightmares (OR 1.48) than those who worked.

When compared with those reporting good or very good self-perceived health, those with average or worse health were at higher risk of adverse changes in their economic situation (OR 1.53), increased loneliness (OR 2.05), and disputes and conflicts within the family (OR 1.67), decrease in feelings of hope for the future (OR 1.80), and increase in sleeping difficulties and nightmares (OR 3.13).

## Discussion

This study explored the sex differences in the psychosocial and economic impact of restrictive measures during COVID-19 in the migrant origin population compared with the general Finnish population. The results show that: (a) Both men and women of migrant origin reported adverse changes in psychosocial and economic impact of COVID-19 more frequently than men and women in the general population. (b) When examining sex differences within the migrant origin population and the general population separately, general population women reported more adverse changes than men in terms of a decrease in the frequency of contact with friends and relatives, increased feelings of loneliness and a decrease in feelings of hope for the future. There were no sex differences between migrant origin men and women in the distribution of any of the studied psychosocial and economic factors. (c) In the migrant origin population, being of migrant origin, rather than sex, increased the odds of reporting more disadvantaged changes in psychosocial and economic impact compared with the general population during COVID-19.

Previous research supports the findings, that migrant origin populations reported more adverse changes in psychosocial and economic impact because of the COVID-19 pandemic and its restrictive measures. This can be explained by the pre-existing vulnerabilities in migrant origin populations that the COVID-19 pandemic had amplified [21]. The impact of migration experience [22], difficulties accessing health services and health-related information [23], as well as experiences of social exclusion and discrimination [24], have been demonstrated to have a negative impact and to create vulnerabilities in psychosocial health and wellbeing, as well as on the socioeconomic situation. According to the European Commission, most of the migrant workers are more likely to work in low-paid, temporary employment and are less compliant to teleworking, compared with the general population [25]. Migrant workers were also more likely than the general population to experience income loss and economic hardship due to the government imposed restrictive measures during COVID-19 [26].

Gendered differences and inequalities between the sexes have been observed already prior to the COVID-19 pandemic, reflected in greater psychosocial and socioeconomic disadvantage. This could be due to a variety of factors, including social isolation, economic strain, and increased responsibilities for caregiving and household tasks during the pandemic, which have reinforced traditional gender roles and labour market inequalities [27]. However, the differences in results between the studied population groups by sex were not consistently in line with the previous research, the gendered differences being adverse especially for women. In our sample, education and employment levels and the language skills of the migrant origin participants were similar to those of the general population reference group, including among women. Therefore, a certain degree of selectivity of the sample may be one of the reasons why we did not see significant gender differences in migrant origin populations, in relation to the general population, even though population sample weights were used to account for non-response. Research on the gendered impact specifically in migrant origin populations and associated factors should be explored further.

It is important to note that it is not migrant origin, nor sex in itself, that predispose to social disadvantage, but rather the complex interaction and accumulation of different intersecting socioeconomic factors, power relations and lived experiences [28]. These intersecting differences can be, for example, a person's age, gender, sexuality, origin, state of health and conviction. These different dimensions and their intersections have an impact on the individual’s position in the society. Therefore, it is important to look at the health, wellbeing and integration of the migrant populations and related political decision making from an intersectional perspective [29]. The findings of this study emphasise the importance of identifying and addressing the vulnerabilities of different population groups effectively to plan and implement measures aimed at reducing the adverse societal impact during future crises. By recognising the specific challenges faced by migrant origin populations, policymakers and stakeholders can develop targeted strategies and interventions to mitigate the negative consequences of such events and promote greater social equity and resilience.

There is a need for strengthened operating systems and policy responses that take into account the specific needs of people in vulnerable positions such as migrant origin populations during serious disruptions and emergencies. This could include measures to provide support and strengthen the coping of those individuals experiencing multiple health and wellbeing impairing factors. Overall, there is a need for further research to understand the complex ways in which the COVID-19 pandemic has impacted the gendered role in migrant origin populations, particularly about the intersectional nature of public health research.

### Strengths and limitations

This study contributes to our knowledge on the gendered impact of COVID-19 among migrant origin populations, as there is a scarcity of previous studies on the topic. Significant strengths of the study are the population-based study design and availability of data for the general Finnish population, as well as the use of standardised questionnaires and the comparable study design across the two surveys. A notable benefit is the availability of self-reported health and wellbeing-related information in both samples, enabling the consideration of the self-reported impact on psychosocial and economic impact, which would not have been attainable solely through registers. The statistical method of interaction effect, studied between the sample and sex, is a significant strength of this study, which allowed the capture of differential effects and improved statistical efficiency than more frequently used sex stratification. Data were collected with multilingual questionnaires, supplemented by multilingual interviews conducted by trained fieldwork personnel. This most likely reduced the language and literacy barriers to participation.

This study has some limitations that should be considered when interpreting the findings. First, the reliance on a single time point for data collection poses a limitation on the study. It is challenging to establish causality or examine the temporal dynamics of the variables under investigation. This study took place in the first year of the COVID-19 pandemic, therefore long-term effects on the impact on psychosocial and economic factors in future studies should be further investigated. Second, the migrant origin population in the current sample, particularly women, had a relatively high level of education, employment and Finnish/Swedish language skills, demonstrating a lower degree of social disadvantage compared with women in the general population than in some previous studies. This may represent a certain degree of selectivity of the sample, even though population sampling weights were used in the current study to account of non-response. It is, nonetheless, possible that those persons of migrant origin, and particularly women, who belonged to less advantaged socioeconomic groups may have chosen or could not participate in the study due to a particular strain of COVID-19 or for other reasons.

## Conclusion

In conclusion, this study examined the psychosocial and economic impact of restrictive measures during COVID-19 on migrant origin populations compared with a nationally representative sample of the general Finnish population. The results indicate that individuals of migrant origin were more likely to report experiencing a greater number of disadvantaged changes in terms of both psychosocial and economic impact during the COVID-19 pandemic when compared with the general population. This suggests that factors related to one’s migrant origin may contribute to increased vulnerability in the face of crises such as the COVID-19 pandemic. Differences in the impact of restrictive measures during COVID-19 between sexes within the migrant origin population were not consistent. This suggests that the psychosocial and economic effects of the pandemic were not influenced solely by gender within this specific group. Further research is needed to explore the complex interactions between sex, migrant origin, and the impact of restrictive measures during COVID-19 in order to gain a more comprehensive understanding of the underlying factors. Promoting social equity among women is likely to reduce gendered inequalities among men and women in all population groups.

## Supplemental Material

sj-docx-1-sjp-10.1177_14034948241235245 – Supplemental material for Psychosocial and economic impact of COVID-19 pandemic by sex among migrant populations compared with general Finnish population: a population-based study Supplemental material, sj-docx-1-sjp-10.1177_14034948241235245 for Psychosocial and economic impact of COVID-19 pandemic by sex among migrant populations compared with general Finnish population: a population-based study by Sanna Nykänen, Regina García-Velázquez, Anu E Castaneda, Päivikki Koponen, Laura Musta and Natalia Skogberg in Scandinavian Journal of Public Health

sj-docx-2-sjp-10.1177_14034948241235245 – Supplemental material for Psychosocial and economic impact of COVID-19 pandemic by sex among migrant populations compared with general Finnish population: a population-based study Supplemental material, sj-docx-2-sjp-10.1177_14034948241235245 for Psychosocial and economic impact of COVID-19 pandemic by sex among migrant populations compared with general Finnish population: a population-based study by Sanna Nykänen, Regina García-Velázquez, Anu E Castaneda, Päivikki Koponen, Laura Musta and Natalia Skogberg in Scandinavian Journal of Public Health

sj-docx-3-sjp-10.1177_14034948241235245 – Supplemental material for Psychosocial and economic impact of COVID-19 pandemic by sex among migrant populations compared with general Finnish population: a population-based study Supplemental material, sj-docx-3-sjp-10.1177_14034948241235245 for Psychosocial and economic impact of COVID-19 pandemic by sex among migrant populations compared with general Finnish population: a population-based study by Sanna Nykänen, Regina García-Velázquez, Anu E Castaneda, Päivikki Koponen, Laura Musta and Natalia Skogberg in Scandinavian Journal of Public Health
