# Supplementary material for: Psychosocial and economic impact of COVID-19 pandemic by sex among migrant populations compared with general Finnish population: a population-based study
Source: Scand J Public Health. 2024 Mar 27;52(3):360–9. doi: 10.1177/14034948241235245 (PMC11067388; doi:10.1177/14034948241235245)
Supplement: sj-docx-1-sjp-10.1177_14034948241235245 – Supplemental material for Psychosocial and economic impact of COVID-19 pandemic by sex among migrant populations compared with general Finnish population: a population-based study [file sj-docx-1-sjp-10.1177_14034948241235245.docx]

**Supplementary Table 2.** Logistic regression model estimates for Model 1.

| **Model 1.** | **Contact with friends and relatives**  OR (95% CI) p-value | **Loneliness**  OR (95% CI) p-value | **Disputes and conflicts within the family**  OR (95% CI) p-value | **Hope for the future**  OR (95% CI) p-value | **Sleeping difficulties, nightmares**  OR (95% CI) p-value | **Economic situation weakened**  OR (95% CI) p-value |
| --- | --- | --- | --- | --- | --- | --- |
| **Sample**  General  Foreign | Ref.  1.02 (0.80-1.30) | Ref.  1.95 (1.51-2.53) ** | Ref.  1.26 (0.91-1.75) | Ref.  1.79 (1.41-2.28) ** | Ref.  2.270 (1.55-3.32) ** | Ref.  5.76 (4.21-7.88) ** |
| **Sex**  Male  Female | Ref.  1.24 (1.00-1.52) * | Ref.  2.04 (1.61-2.57) ** | Ref.  1.26 (0.93-1.72) | Ref.  1.69 (1.35-2.11) ** | Ref.  1.71 (1.19-2.44) * | Ref.  1.47 (0.98-2.21) |
| **Sample x Sex**  Foreign Female | 0.80 (0.58-1.09) | 0.56 (0.40-0.78) * | 0.83 (0.53-1.30) | 0.60 (0.43-0.83) * | 0.80 (0.50-1.29) | 0.52 (0.31-0.86) * |
| **Age** 20-34  35-49  50-66 | Ref.  1.08 (0.87-1.35)  1.25 (0.99-1.58) | Ref.  0.80 (0.64-1.00) *  0.60 (0.48-0.75) ** | Ref.  0.97 (0.73-1.29)  0.58 (0.43-0.79) ** | Ref.  0.92 (0.74-1.14)  0.96 (0.74-1.15) | Ref.  0.91 (0.65-1.27)  0.97 (0.70-1.36) | Ref.  0.80 (0.59-1.08)  0.60 (0.43-0.83) ** |
| OR = odds ratio; CI = 95 % confidence interval; Ref. = Reference group. Results are reported in weighted values.  * = p-value <0.05, **= p-value <.001  Model 1. adjusted with age and sex. | | | | | | |
