# Supplementary material for: Psychosocial and economic impact of COVID-19 pandemic by sex among migrant populations compared with general Finnish population: a population-based study
Source: Scand J Public Health. 2024 Mar 27;52(3):360–9. doi: 10.1177/14034948241235245 (PMC11067388; doi:10.1177/14034948241235245)
Supplement: sj-docx-2-sjp-10.1177_14034948241235245 – Supplemental material for Psychosocial and economic impact of COVID-19 pandemic by sex among migrant populations compared with general Finnish population: a population-based study [file sj-docx-2-sjp-10.1177_14034948241235245.docx]

**Supplementary Table 3.** Logistic regression model estimates for Model 2.

| **Model 2.** | **Contact with friends and relatives**  OR (95% CI) p-value | **Loneliness**  OR (95% CI) p-value | **Disputes and conflicts within the family**  OR (95% CI) p-value | **Hope for the future**  OR (95% CI) p-value | **Sleeping difficulties, nightmares**  OR (95% CI) p-value | **Economic situation weakened**  OR (95% CI) p-value |
| --- | --- | --- | --- | --- | --- | --- |
| **Sample**  General  Foreign | Ref.  1.02 (0.80-1.30) | Ref.  1.83 (1.42-2.37) ** | Ref.  1.25 (0.90-1.73) | Ref.  1.78 (1.40-2.27) ** | Ref.  2.16 (1.48-3.15) ** | Ref.  5.61 (4.12-7.65) ** |
| **Sex**  Male  Female | Ref.  1.17 (0.94-1.44) | Ref.  1.87 (1.47-2.38) ** | Ref.  1.23 (0.89-1.70) | Ref.  1.62 (1.29-2.03) * | Ref.  1.60 (1.11-2.31) * | Ref.  1.41 (0.92-2.16) |
| **Sample x Sex**  Foreign Female | 0.84 (0.61-1.16) | 0.58 (0.45-0.82) * | 0.88 (0.56-1.39) | 0.63 (0.45-0.87) * | 0.86 (0.53-1.39) | 0.52 (0.31-0.86) * |
| **Age** 20-34  35-49  50-66 | Ref.  1.11 (0.89-1.38)  1.24 (0.98-1.57) | Ref.  0.80 (0.64-1.01)  0.63 (0.50-0.80) ** | Ref.  0.91 (0.68-1.23)  0.56 (0.41-0.77) ** | Ref.  0.89 (0.71-1.12)  0.99 (0.79-1.25) | Ref.  0.94 (0.68-1.32)  0.97 (0.68-1.37) | Ref.  0.85 (0.63-1.16)  0.50 (0.35-0.70) ** |
| **Education**  Basic level or less  Secondary  Higher | Ref.  1.56 (1.19-2.05) **  2.34 (1.78-3.09) ** | Ref.  0.96 (0.71-1.31)  1.73 (1.28-2.35) ** | Ref.  1.35 (0.87-2.11)  1.74 (1.12-2.70) * | Ref.  1.36 (1.01-1.84) *  1.98 (1.47-2.67) ** | Ref.  1.36 (0.90-2.05)  1.70 (1.15-2.52) ** | Ref.  1.30 (0.85-1.99)  0.97 (0.63-1.49) |
| **Economic activity**  Working  Student  Other | Ref.  0.62 (0.41-0.93) *  0.93 (0.78-1.12) | Ref.  1.12 (0.75-1.68)  1.45 (1.19-1.77) ** | Ref.  0.75 (0.48-1.17)  0.99 (0.76-1.29) | Ref.  1.06 (0.75-1.49)  0.98 (0.80-1.19) | Ref.  1.33 (0.77-2.31)  1.68 (1.26-2.24) ** | Ref.  1.59 (1.01-2.49) *  3.05 (2.31-4.04) ** |
| OR = odds ratio; CI = 95 % confidence interval; Ref. = Reference group. Results are reported in weighted values.  * = p-value <0.05, **= p-value <.001  Model 2. adjusted with age, sex, education and economic activity. | | | | | | |
