# Supplementary material for: Psychosocial and economic impact of COVID-19 pandemic by sex among migrant populations compared with general Finnish population: a population-based study
Source: Scand J Public Health. 2024 Mar 27;52(3):360–9. doi: 10.1177/14034948241235245 (PMC11067388; doi:10.1177/14034948241235245)
Supplement: sj-docx-3-sjp-10.1177_14034948241235245 – Supplemental material for Psychosocial and economic impact of COVID-19 pandemic by sex among migrant populations compared with general Finnish population: a population-based study [file sj-docx-3-sjp-10.1177_14034948241235245.docx]

**Supplementary Table 1.** Psychosocial and economic factors prevalence rates and 95% confidence intervals (CI) by country groups, men and women.

| **Men** | **Russia, former Soviet Union, n=** | **Rest of Europe, North America, and Oceania,**  **n=632** | **Africa and Middle East, n=483** | **Asia and Latin America, n=281** | **Born abroad**  **total, Men** | **General population, Men** |
| --- | --- | --- | --- | --- | --- | --- |
| **Contact with friends and relatives**  Increased  Decreased  No Effect/Does not apply | 9.0 (4.1-13.8)  58.5 (50.2-66.8)  32.5 (24.5-40.5) | 18.6 (12.9-24.3)  57.8 (50.7-64.8)  23.6 (17.5-29.7) | 19.7 (13.0-26.4)  51.9 (43.3-60.5)  28.4 (20.3-36.5) | 17.2 (9.5-24.8)  63.3 (54.1-72.4)  19.6 (13.0-26.2) | 16.9 (13.7-20.2)  57.3 (53.1-61.5)  25.8 (22.1-29.4) | 4.5 (3.4-5.7)  56.8 (52.8-60.8)  38.7 (34.7-42.8) |
| **Loneliness**  Increased  Decreased  No Effect/Does not apply | 9.0 (4.1-13.8)  58.5 (50.2-66.8)  32.5 (24.5-40.5) | 28.7 (22.6-34.8)  6.1 (2.5-9.7)  65.2 (58.7-71.8) | 41.5 (32.9-50.0)  10.2 (5.7-14.7)  48.3 (39.7-57.0) | 4.7 (37.6-56.4)  7.4 (2.5-12.3)  45.6 (36.4-54.8) | 33.7 (29.8-37.6)  6.7 (4.6-8.7)  59.7 (55.6-63.7) | 20.8 (17.8-23.9)  1.5 (0.9-2.2)  77.7 (74.6-80.8) |
| **Disputes and conflicts within the family**  Increased  Decreased  No Effect/Does not apply | 5.7 (2.7-8.6)  5.1 (1.2-8.9)  89.3 (84.5-94.0) | 18.5 (12.8-24.2)  5.1 (1.9-8.4)  76.3 (70.2-82.5) | 14.4 (8.8-20.0)  5.1 (1.9-8.4)  76.1 (69.0-83.3) | 15.7 (8.9-22.5)  7.8 (2.3-13.4)  76.5 (68.3-84.7) | 14.7 (11.7-17.6)  6.7 (4.5-8.9)  78.7 (75.2-82.1 | 12.0 (9.7-14.3)  2.2 (1.3-3.1)  85.8 (83.4-88.1) |
| **Hope for the future**  Increased  Decreased  No Effect/Does not apply | 10.3 (5.1-15.5)  32.0 (24.6-39.3)  57.7 (49.6-65.8) | 16.6 (11.7-21.5)  33.8 (27.2-40.5)  49.5 (42.5-56.6) | 25.2 (17.2-33.3)  36.1 (28.4-43.9)  38.6 (30.2-47.1) | 5.9 (4.2-7.6)  48.4 (38.8-57.9)  30.6 (21.2-40.1) | 18.5 (15.1-21.8)  36.6 (32.7-40.5)  44.9 (40.7-49.1) | 5.9 (4.2-7.7)  24.4 (21.3-27.5)  69.7 (66.4-73.0) |
| **Sleeping difficulties, nightmares**  Increased  Decreased  No Effect/Does not apply | 6.4 (3.0-9.8)  2.4 (0.0-5.0)  91.2 (87.0-95.4) | 13.8 (9.2-18.3)  1.4 (0.2-2.6)  84.9 (80.2-89.6) | 21.8 (14.2-29.5)  8.4 (3.0-13.9)  69.7 (61.3-78.2) | 17.1 (9.6-24.6)  5.0 (0.2-9.9)  77.9 (69.5-86.3) | 15.1 (12.0-18.2)  4.0 (2.2-5.9)  80.9 (77.4-84.3) | 7.3 (5.3-9.3)  0.8 (0.4-1.3)  91.9 (89.8-93.9) |
| **Economic situation weakened**  No  Yes | 85.1 (79.5-90.6)  14.9 (9.4-20.5) | 82.2 (76.7-87.7)  17.8 (12.3-23.3) | 70.4 (62.4-78.3)  29.6 (21.7-37.6) | 66.1 (56.6-75.6)  33.9 (24.4-43.4) | 76.9 (73.2-80.5)  23.1 (19.5-26.8) | 94.9 (93.8-96.1)  5.1 (3.9-6.2) |
| **Women** | **Russia, former Soviet Union,**  **n=464** | **Rest of Europe, North America and Oceania, n=632** | **Africa and Middle East, n=483,** | **Asia and Latin America, n=369** | **Born abroad**  **total, Women, n=1562** | **General population, Women, n=1780** |
| **Contact with friends and relatives**  Increased  Decreased  No Effect/Does not apply | 11.2 (7.5-14.9)  61.7 (55.3-68.2)  27.1 (20.9-33.3) | 18.8 (13.6-23.9)  58.9 (52.0-65.8)  22.3 (16.1-28.5) | 28.4 (16.5-40.3)  46.4 (33.2-59.6)  25.2 (12.6-37.8) | 18.7 (13.6-23.7)  56.1 (49.2-62.9)  25.2 (19.2-31.3) | 18.1 (15.1-21.2)  56.9 (52.9-60.9)  24.9 (21.2-28.6) | 10.2 (7.8-12.5)  61.9 (58.4-65.4)  28.0 (24.8-31.2) |
| **Loneliness**  Increased  Decreased  No Effect/Does not apply | 29.9 (24.5-35.4)  2.9 (0.4-5.4)  67.1 (61.3-72.9) | 39.6 (32.9-46.3)  2.8 (0.6-5.0)  57.6 (50.8-64.4) | 35.8 (23.1-48.6)  2.8 (0.6-5.0)  52.0 (38.8-65.2) | 41.6 (34.7-48.5)  9.5 (4.7-14.39  48.9 (41.9-56.0) | 36.5 (32.7-40.3)  6.0 (3.8-8.2)  57.5 (53.6-61.4) | 34.6 (31.1-37.8)  3.0 (1.4-4.5)  62.5 (59.2-65.7) |
| **Disputes and conflicts within the family**  Increased  Decreased  No Effect/Does not apply | 13.9 (9.3-18.5)  2.2 (1.1-3.4)  83.8 (79.1-88.6) | 14.1 (9.5-18.7)  5.5 (1.7-9.4)  80.4 (74.7-86.0) | 19.5 (9.2-29.9)  12.1 (3.2-20.9)  68.4 (56.2-80.6) | 15.4 (10.2-20.5)  8.0 (3.6-12.4)  76.6 (70.3-82.9) | 15.3 (12.4-18.2)  6.2 (4.0-8.5)  78.5 (75.1-81.9) | 14.6 (1.2-17.2)  3.2 (2.3-4.1)  82.1 (79.4-84.9) |
| **Hope for the future**  Increased  Decreased  No Effect/Does not apply | 14.9 (10.3-19.6)  32.7 (2.7-38.4)  52.4 (4.6-58.7) | 15.8 (11.0-20.5)  44.1 (37.3-50.9)  40.1 (33.2-47.0) | 30.9 (18.0-43.8)  33.4 (21.6-45.2)  35.7 (22.9-48.5) | 26.5 (20.2-32.8)  35.9 (29.4-42.3)  37.6 (30.7-44.6) | 20.7 (17.2-24.1)  36.9 (33.2-40.6)  42.4 (38.5-46.4) | 6.9 (5.6-8.2)  35.2 (31.9-38.5)  57.9 (54.5-61.3) |
| **Sleeping difficulties, nightmares**  Increased  Decreased  No Effect/Does not apply | 14.4 (10.3-18.5)  1.2 (2.0-2.2)  84.3 (80.1-88.5) | 18.1 (13.4-22.8)  3.1 (5.0-5.7)  78.8 (73.7-84.0) | 29.2 (16.8-41.6)  7.6 (0.00-15.5)  63.2 (50.0-76.4) | 21.2 (15.4-26.9)  2.9 (0.8-4.9)  76.0 (70.0-81.9) | 19.6 (16.4-22.8)  3.2 (1.5-5.0)  77.1 (73.7-80.6) | 11.8 (9.3-14.4)  2.0 (3.0-3.7)  86.2 (83.3-89.1) |
| **Economic situation weakened**  No  Yes | 85.0 (81.4-88.6)  15.0 (11.4-18.6) | 83.1 (77.6-88.6)  16.9 (11.4-22.4) | 22.3 (9.8-34.9)  77.7 (65.1-90.2) | 77.2 (71.2-83.3)  22.8 (16.7-28.8) | 81.4 (78.1-84.6)  18.6 (15.4-21.9) | 92.8 (90.5-95.1)  7.2 (4.9-9.5) |

**Supplementary Table 2.** Logistic regression model estimates for Model 1.

| **Model 1.** | **Contact with friends and relatives**  OR (95% CI) p-value | **Loneliness**  OR (95% CI) p-value | **Disputes and conflicts within the family**  OR (95% CI) p-value | **Hope for the future**  OR (95% CI) p-value | **Sleeping difficulties, nightmares**  OR (95% CI) p-value | **Economic situation weakened**  OR (95% CI) p-value |
| --- | --- | --- | --- | --- | --- | --- |
| **Sample**  General  Foreign | Ref.  1.02 (0.80-1.30) | Ref.  1.95 (1.51-2.53) ** | Ref.  1.26 (0.91-1.75) | Ref.  1.79 (1.41-2.28) ** | Ref.  2.270 (1.55-3.32) ** | Ref.  5.76 (4.21-7.88) ** |
| **Sex**  Male  Female | Ref.  1.24 (1.00-1.52) * | Ref.  2.04 (1.61-2.57) ** | Ref.  1.26 (0.93-1.72) | Ref.  1.69 (1.35-2.11) ** | Ref.  1.71 (1.19-2.44) * | Ref.  1.47 (0.98-2.21) |
| **Sample x Sex**  Foreign Female | 0.80 (0.58-1.09) | 0.56 (0.40-0.78) * | 0.83 (0.53-1.30) | 0.60 (0.43-0.83) * | 0.80 (0.50-1.29) | 0.52 (0.31-0.86) * |
| **Age** 20-34  35-49  50-66 | Ref.  1.08 (0.87-1.35)  1.25 (0.99-1.58) | Ref.  0.80 (0.64-1.00) *  0.60 (0.48-0.75) ** | Ref.  0.97 (0.73-1.29)  0.58 (0.43-0.79) ** | Ref.  0.92 (0.74-1.14)  0.96 (0.74-1.15) | Ref.  0.91 (0.65-1.27)  0.97 (0.70-1.36) | Ref.  0.80 (0.59-1.08)  0.60 (0.43-0.83) ** |
| OR = odds ratio; CI = 95 % confidence interval; Ref. = Reference group. Results are reported in weighted values.  * = p-value <0.05, **= p-value <.001  Model 1. adjusted with age and sex. | | | | | | |

**Supplementary Table 3.** Logistic regression model estimates for Model 2.

| **Model 2.** | **Contact with friends and relatives**  OR (95% CI) p-value | **Loneliness**  OR (95% CI) p-value | **Disputes and conflicts within the family**  OR (95% CI) p-value | **Hope for the future**  OR (95% CI) p-value | **Sleeping difficulties, nightmares**  OR (95% CI) p-value | **Economic situation weakened**  OR (95% CI) p-value |
| --- | --- | --- | --- | --- | --- | --- |
| **Sample**  General  Foreign | Ref.  1.02 (0.80-1.30) | Ref.  1.83 (1.42-2.37) ** | Ref.  1.25 (0.90-1.73) | Ref.  1.78 (1.40-2.27) ** | Ref.  2.16 (1.48-3.15) ** | Ref.  5.61 (4.12-7.65) ** |
| **Sex**  Male  Female | Ref.  1.17 (0.94-1.44) | Ref.  1.87 (1.47-2.38) ** | Ref.  1.23 (0.89-1.70) | Ref.  1.62 (1.29-2.03) * | Ref.  1.60 (1.11-2.31) * | Ref.  1.41 (0.92-2.16) |
| **Sample x Sex**  Foreign Female | 0.84 (0.61-1.16) | 0.58 (0.45-0.82) * | 0.88 (0.56-1.39) | 0.63 (0.45-0.87) * | 0.86 (0.53-1.39) | 0.52 (0.31-0.86) * |
| **Age** 20-34  35-49  50-66 | Ref.  1.11 (0.89-1.38)  1.24 (0.98-1.57) | Ref.  0.80 (0.64-1.01)  0.63 (0.50-0.80) ** | Ref.  0.91 (0.68-1.23)  0.56 (0.41-0.77) ** | Ref.  0.89 (0.71-1.12)  0.99 (0.79-1.25) | Ref.  0.94 (0.68-1.32)  0.97 (0.68-1.37) | Ref.  0.85 (0.63-1.16)  0.50 (0.35-0.70) ** |
| **Education**  Basic level or less  Secondary  Higher | Ref.  1.56 (1.19-2.05) **  2.34 (1.78-3.09) ** | Ref.  0.96 (0.71-1.31)  1.73 (1.28-2.35) ** | Ref.  1.35 (0.87-2.11)  1.74 (1.12-2.70) * | Ref.  1.36 (1.01-1.84) *  1.98 (1.47-2.67) ** | Ref.  1.36 (0.90-2.05)  1.70 (1.15-2.52) ** | Ref.  1.30 (0.85-1.99)  0.97 (0.63-1.49) |
| **Economic activity**  Working  Student  Other | Ref.  0.62 (0.41-0.93) *  0.93 (0.78-1.12) | Ref.  1.12 (0.75-1.68)  1.45 (1.19-1.77) ** | Ref.  0.75 (0.48-1.17)  0.99 (0.76-1.29) | Ref.  1.06 (0.75-1.49)  0.98 (0.80-1.19) | Ref.  1.33 (0.77-2.31)  1.68 (1.26-2.24) ** | Ref.  1.59 (1.01-2.49) *  3.05 (2.31-4.04) ** |
| OR = odds ratio; CI = 95 % confidence interval; Ref. = Reference group. Results are reported in weighted values.  * = p-value <0.05, **= p-value <.001  Model 2. adjusted with age, sex, education and economic activity. | | | | | | |
